# ZnFe_2_O_4_-TiO_2_ Nanoparticles within Mesoporous MCM-41

**DOI:** 10.1100/2012/480527

**Published:** 2012-07-31

**Authors:** Aidong Tang, Yuehua Deng, Jiao Jin, Huaming Yang

**Affiliations:** ^1^School of Chemistry and Chemical Engineering, Central South University, Changsha 410083, China; ^2^Department of Inorganic Materials, School of Resources Processing and Bioengineering, Central South University, Changsha 410083, China

## Abstract

A novel nanocomposite ZnFe_2_O_4_-TiO_2_/MCM-41 (ZTM) was synthesized by a sol-gel method and characterized through X-ray diffraction (XRD), high-resolution transmission electron microscopy (HRTEM), N_2_ adsorption-desorption, Raman spectroscopy, and ultraviolet visible (UV-vis) spectrophotometry. The results confirmed the incorporation of ZnFe_2_O_4_-TiO_2_ nanoparticles inside the pores of the mesoporous MCM-41 host without destroying its integrity. ZnFe_2_O_4_ nanoparticles can inhibit the transformation of anatase into rutile phase of TiO_2_. Incorporation of ZnFe_2_O_4_-TiO_2_ within MCM-41 avoided the agglomeration of nanoparticles and reduced the band gap energy of TiO_2_ to enhance its visible light photocatalytic activity. UV-vis absorption edges of ZTM nanocomposites redshifted with the increase of Zn/Ti molar ratio. The nanocomposite approach could be a potential choice for enhancing the photoactivity of TiO_2_, indicating an interesting application in the photodegradation and photoelectric fields.

## 1. Introduction

TiO_2_ had been widely used in various applications, such as functional ceramics [1, 2], sensor materials [3–5], cosmetic products [6, 7], photocatalyst [8–10], high grade coatings [11–13], pigment in the paint, and paper and pulp industry [14, 15]. As an interesting photocatalyst, TiO_2_ had attracted much attention due to high efficiency with low cost, chemical inertness, and photostability. Different kinds of TiO_2_ were obtained through several procedures: porous TiO_2_ cryogel fibers through unidirectional freezing of hydrogel [16], wormhole-like mesoporous TiO_2_ by chemical vapor deposition [17], hollow TiO_2_ microspheres obtained by a single-step synthesis in ionic liquids [18], and coral-like TiO_2_ produced by templating polymer gels [17]. However, the widespread use of TiO_2_ was impaired by some defects of its structure, agglomeration easily occurred during the synthesis process and wide band gap (3.2 eV) which requires ultraviolet irradiation for photocatalytic activation.

In order to improve the photocatalytic properties of TiO_2_, effective measurements had been taken into experiment. Doping of transition [19] and noble metals [20, 21] can improve the photocatalytic activity of TiO_2_ to some degree. Zinc ferrite (ZnFe_2_O_4_), with a spinel structure and a relatively small band gap (1.9 eV), has a potential application in the conversion of sunlight. However, because of the lower valence band potential and poor property in photoelectric conversion, ZnFe_2_O_4_ cannot be directly used in the photocatalytic destruction. It was demonstrated that ZnFe_2_O_4_ doping extend the adsorption spectrum to the longer wavelength, this material had high utility of sunlight, high photoactivity, and high efficiency of photoelectric conversion [22–24].

Meanwhile, considering that mesoporous molecular sieves, MCM-41, which possesses a regular hexagonal array of uniform pore openings, we propose that the uniform ordered channels of MCM-41 may be able to control the particle size of TiO_2_ and efficiently prevent particles from agglomeration. It was a new route to keep nanoparticles inside the pores of MCM-41 material [25]. There are many researches focused on the substitution of silicon by metals such as Ti [26] and Ni [27] and incorporating nanoparticles within the pores of mesoporous materials, including gold nanoparticles within mesoporous silica [28], MCM-41 modified SnO_2_ [29], and MCM-48 containing TiO_2_ nanoparticles [30], all of which can enhance the properties of the nanoparticles or the incorporated hosts.

In this paper, we demonstrate a novel route to improve the properties of TiO_2_ through the addition of ZnFe_2_O_4_ and incorporating the ZnFe_2_O_4_-TiO_2_ nanoparticles into the channels of mesoporous MCM-41. The effect of ZnFe_2_O_4_ addition on the structure and the properties of ZnFe_2_O_4_-TiO_2_ and ZnFe_2_O_4_-TiO_2_/MCM-41 composites are also investigated. The composites were in detail characterized by XRD, TG-DSC, TEM, BET, Raman, and UV-vis spectroscopy.

## 2. Experimental

### 2.1. Materials Synthesis

MCM-41 mesoporous materials were prepared according to our previous work [31]. TiO_2_/MCM-41 composite (molar ratio of Ti/Si = 0.4) was obtained using tetrabutyl titanate as Ti source. The synthesis procedure of TiO_2_/MCM-41 composite was as follows: a certain amount of tetrabutyl titanate was added to 40 mL ethanol with uniform stirring. Diethanolamine, used as catalyst and stabilizing agent, was added and constantly stirred for 0.5 h to form a solution S. 1.8 g MCM-41 was added to the above solution S and stirred for 10 min. A mixture of water and ethanol was further dropped, stirred for 4 h and aged for 24 h to obtain a gel. The gel was dried at 80°C overnight and calcined at 600°C to produce TiO_2_/MCM-41 composites. Pure TiO_2_ was synthesized according to this procedure without the addition of MCM-41. The synthesis procedure of ZnFe_2_O_4_-TiO_2_ with different Zn/Ti molar ratio was similar to the above procedure as TiO_2_. Certain amount of ferric nitrate and zinc acetate (molar ratio of Zn/Fe = 0.5) were dissolved in an ethanol solution to obtain a uniform precursor. The precursor was added to the solution S with different Zn/Ti molar ratios (1%, 3%, 5%, and 7%, denoting the corresponding product ZnFe_2_O_4_-TiO_2_ as ZT1, ZT3, ZT5, and ZT7, resp.). When MCM-41 was simultaneously added to the above solution S according to Ti/Si molar ratio of 0.4, the final product ZnFe_2_O_4_-TiO_2_/MCM-41 was correspondingly indicated as ZTM1, ZTM3, ZTM5, and ZTM7, respectively.

### 2.2. Characterization

X-ray diffraction (XRD) was carried out using a Bruker D8 advance with Cu K*α* radiation (*λ* = 0.15406 nm) over the scanning range 2*θ* = 1°~10° for small angle XRD (SAXRD) at a voltage of 40 kV and 300 mA and 2*θ* = 1°~10° for wide angle XRD (WAXRD) at a voltage of 40 kV and 200 mA both with a step width of 0.0085°. Nitrogen gas adsorption-desorption isotherms were measured at 77 K using an ASAP 2020 unit. Prior to the sorption experiment, the samples were vacuum-dried at 200°C for 10 h. The specific surface area and pore size distribution were calculated by the Brunauer-Emmett-Teller (BET) method and the Barrett-Joyner-Halenda (BJH) method using the adsorption-desorption isotherms, respectively. The total pore volume was obtained from the maximum amount of nitrogen gas adsorbed at partial pressure (*P*/*P*_0_ = 0.999). A Tecnai G220 AEM electron microscope operating at accelerating voltages up to 200 kV was used for the high-resolution TEM (HRTEM) studies. Samples were prepared by suspending MCM-41 material with or without TiO_2_ nanoparticles in ethanol followed by sonication for 15 min in an ultrasonic bath. The suspension was dripped onto a carbon-coated copper grid and allowed to dry, respectively. Ultraviolet visible (UV-vis) spectrophotometry spectra were collected on a SHIMADZU UV-2450 spectrophotometer at room temperature, and the detection range of wavelength is from 190 nm to 700 nm. The Raman spectra were obtained using Renishaw InVia Raman system which can extend to 100 cm^−1^. The 514 nm line of an Argon laser was used as the excitation source.

## 3. Results and Discussion

**Figure 1:**
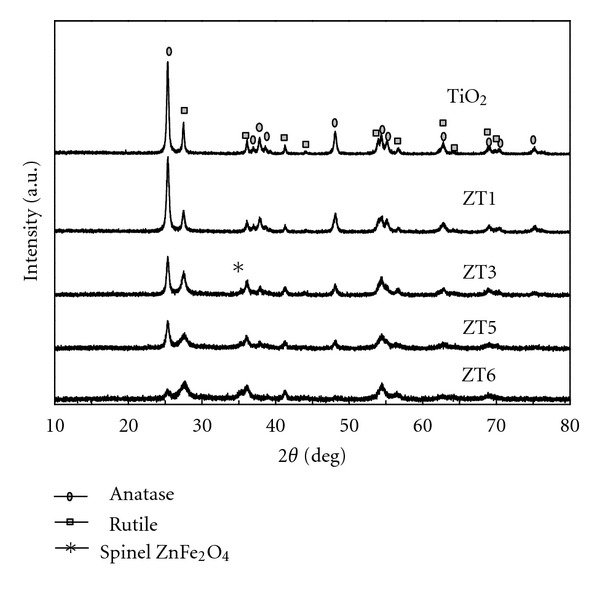
WAXRD patterns of pure TiO_2_ and ZT series samples.

**Figure 2:**
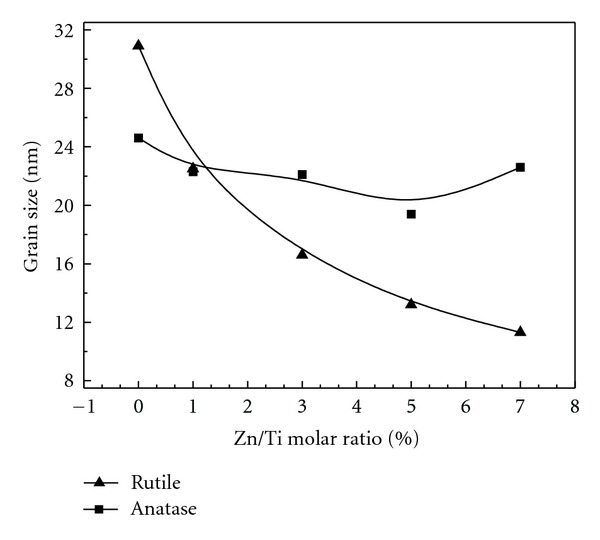
Variation of grain sizes of anatase and rutile TiO_2_ in ZT series samples.

XRD analysis can provide detailed information on crystallite structure characteristics. WAXRD patterns of pure TiO_2_ and ZT series samples showed both anatase and rutile phase of TiO_2_ (Figure 1). The peak intensity of rutile phase decreased with increasing the amount of ZnFe_2_O_4_. The percentage of anatase phase in pure TiO_2_ and ZT series (Zn/Ti from 1% to 7%) were 74%, 76%, 52%, 55%, and 18%, respectively. It was concluded that the addition of ZnFe_2_O_4_ can promote the transformation of TiO_2_ from anatase to rutile phase. Li et al. [32] and Liu et al. [23] obtained similar conclusion from their study. Meanwhile, all the peaks became broadened, suggesting that the grain size decreased with the increasing addition of ZnFe_2_O_4_. A weak diffraction peak due to spinel ZnFe_2_O_4_ phase appeared at the Zn/Ti molar ratio of 3%, indicating the formation of ZnFe_2_O_4_ in the composites, which was in good accordance with the references [33, 34]. The grain sizes of anatase and rutile TiO_2_ phase were calculated using Jade 5.0 (Figure 2) which all decreased with increasing the amount of ZnFe_2_O_4_, ZnFe_2_O_4_ seemed to play an important role in inhibiting the growth of TiO_2_ particles [35, 36]. 

**Figure 3:**
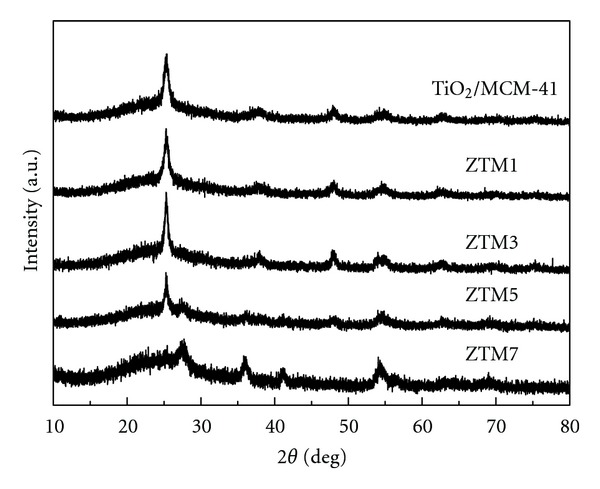
WAXRD patterns of TiO_2_/MCM-41 and ZTM series samples.

Figure 3 showed the WAXRD patterns of MCM-41 incorporated within pure TiO_2_ and ZT nanoparticles. TiO_2_/MCM-41, ZTM1, and ZTM3 only presented the characteristic peaks of anatase TiO_2_, while rutile phase was observed in the WAXRD patterns of the other two composites. The peaks corresponding to anatase and rutile became broadened, which was attributed to the confinement of nanoparticles within MCM-41 pore channel. All the patterns did not show any peaks corresponding to spinel ZnFe_2_O_4_. The percentage of anatase in ZTM5 and ZTM7 were 69% and 28%, respectively, larger than those of ZT5 and ZT7 samples (55% and 18%), suggesting that MCM-41 had an obvious effect on the anatase-rutile phase transformation of TiO_2_.

**Figure 4:**
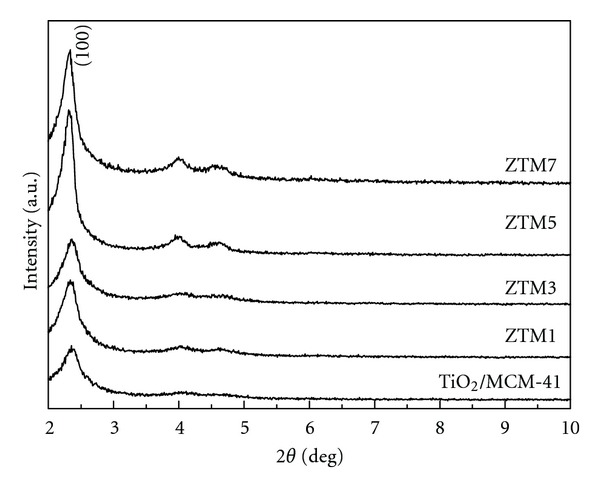
SAXRD patterns of TiO_2_/MCM-41 and ZTM series samples.

All the samples presented three characteristic peaks of typical MCM-41 in their SAXRD patterns (Figure 4), which was in accordance with the previous report [37], indicating that the pore channel of MCM-41 remained well even after the incorporation of nanoparticles. No change in the position of main peak demonstrated that Ti^4+^ ions, Zn^2+^ ions, and Fe^3+^ ions were not incorporated into the framework of MCM-41. The increase in intensity of (100) peak after incorporation demonstrated that ZnFe_2_O_4_ can inhibit the growth of TiO_2_ particles. However, compared with SAXRD pattern of pure MCM-41 [31], the peak intensity of all samples decreased visibly, one can be attributed to the pore filling of the host material, which reduced the scattering contrast between pore walls and pores, thus leading to a decrease in peak intensity [38]; another is possibly related to the loss of sample integrity [30].

**Figure 5:**
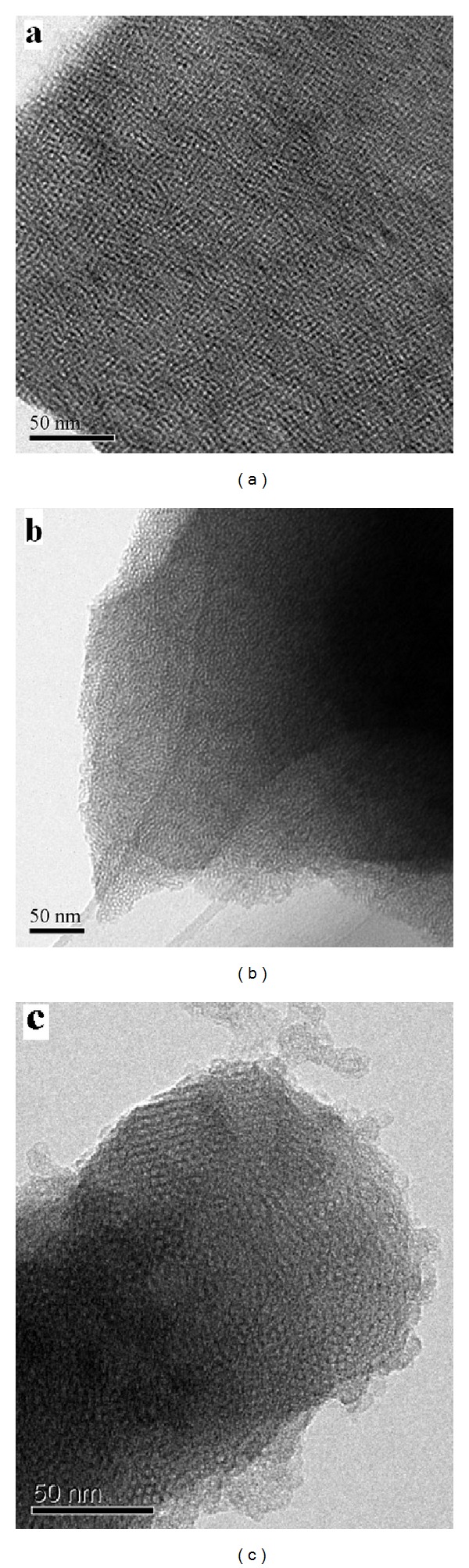
HRTEM images of (a) MCM-41, (b) TiO_2_/MCM-41, and (c) ZTM7 sample.

**Figure 6:**
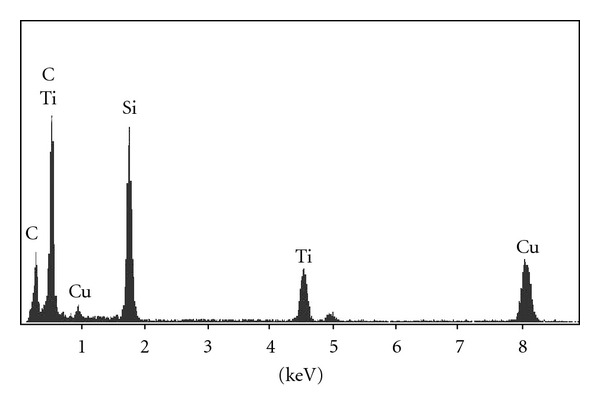
EDS spectrum of TiO_2_/MCM-41 sample.

HRTEM images of MCM-41, TiO_2_/MCM-41, and ZnFe_2_O_4_-TiO_2_/MCM-41 (Zn : Tin = 0.07, ZTM = 7) were collected to find out the location of TiO_2_ (Figure 5), the ordered mesopores of MCM-41 with an average pore size of 3 nm were clearly observed. After incorporated with nanoparticles, the pore channels were maintained well, and no TiO_2_ particles were detected on the surface of MCM-41. EDS spectrum of TiO_2_/MCM-41 (Figure 6) showed the existence of Si, O, and Ti elements with the Ti/Si molar ratio approximatively equal to the experimental value (Ti/Si = 0.4). The HRTEM images of ZTM7 also displayed the ordered hexagonal mesopores without any particles on the surface. Combined with above XRD results, it was concluded that the TiO_2_ or ZT nanoparticles were incorporated into the pore channel of MCM-41.

**Figure 7:**
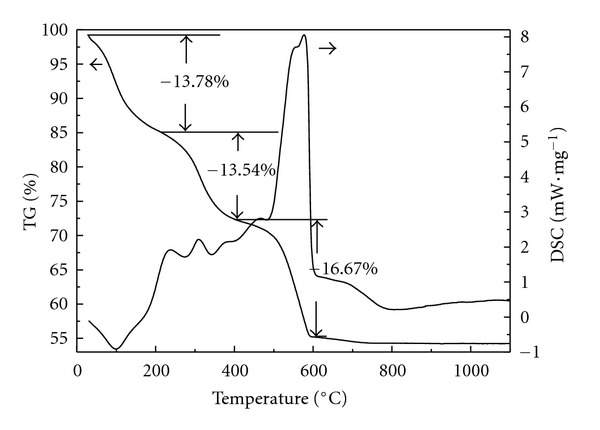
TG-DSC curves of TiO_2_ sample.

**Figure 8:**
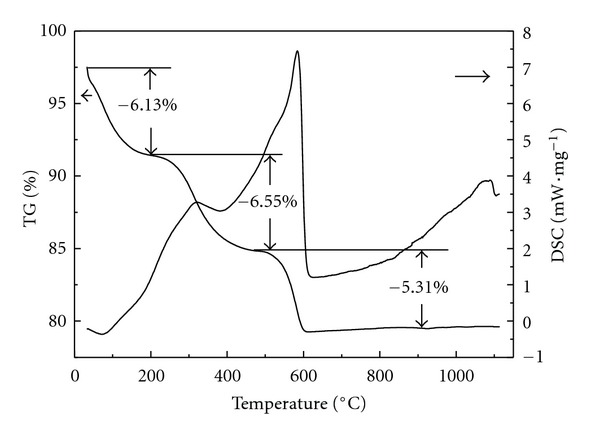
TG-DSC curves of TiO_2_/MCM-41 sample.

**Figure 9:**
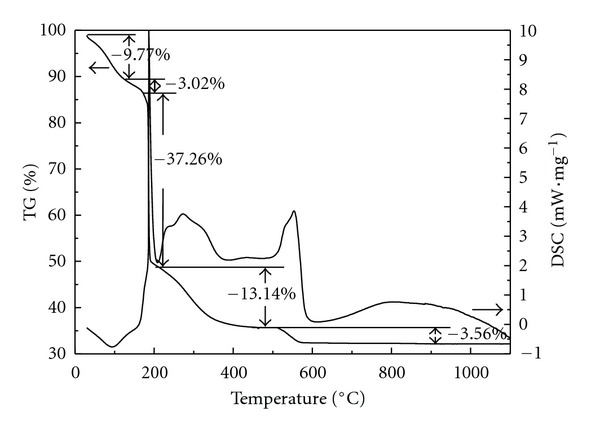
TG-DSC curves of ZT7 sample.

TG-DSC measurements were performed from room temperature to 1100°C to reveal the thermal behavior of three precursors (Figures 7–9). It was well known that the thermal behavior of TiO_2_ usually depended on the chemical composition, preparation condition, and existing phases [39]. An endothermic peak at 99°C with mass loss of 13.78% was due to the desorption of physically adsorbed water (Figure 7). Three small exothermic peaks at 200~400°C were due to the decomposition and oxidation of organic substances as well as the transformation of TiO_2_ from amorphous to anatase phase [40]. Peaks at 400~600°C with mass loss of 16.67% were attributed to the oxidation of residual organic substances and the dehydration of structural water. From the above XRD result, TiO_2_ particles were composed of anatase and rutile phases, therefore, the strong peak at 500~600°C also contained the exothermic peak of phase transformation from anatase to rutile. An exothermic shoulder at 600~800°C could be related to the phase transformation of TiO_2_ from anatase to rutile. The DSC curve of TiO_2_/MCM-41 was much simpler than that of TiO_2_, only one endothermic peak and two exothermic peaks appeared in the DSC curves (Figure 8). The endothermic peak at 70°C was due to the adsorbed water, and the peaks at 323°C and 584°C can be attributed to the oxidation of organic substances and the dehydration of structure water. The whole mass loss of TiO_2_/MCM-41 precursor was 17.99%. The amount of TiO_2_ incorporated to MCM-41 can be calculated from the mass loss, and the result was basically in accordance with the experimentally designed values. Since the XRD showed that TiO_2_/MCM-41 only contained pure anatase phase TiO_2_ (Figure 3), the exothermic area from 800~1000°C was directly related to the phase transformation from anatase to rutile. The DSC curve of ZTM7 appeared as a sharp peak at 187°C with a big mass loss of 37.26% (Figure 9), probably presenting the oxidation of organic substances and the decomposition of nitrate. The peaks at 200~600°C were due to the oxidation of residual organic substances and the dehydration of precursor, accompanying the crystallization of ZnFe_2_O_4_, and the phase transformation of TiO_2_.

**Figure 10:**
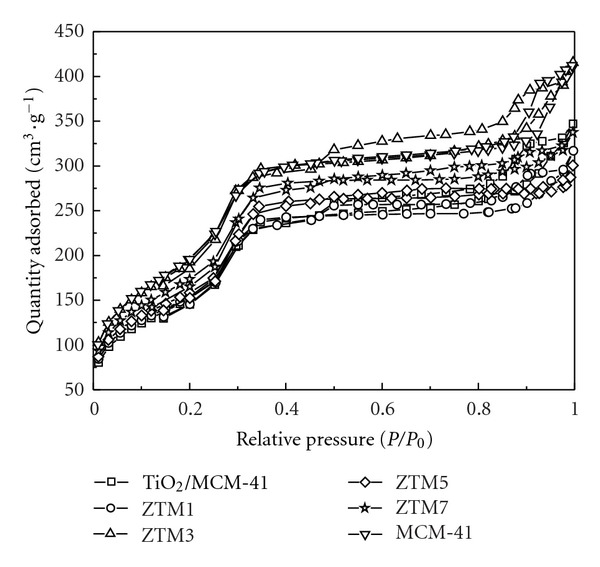
N_2_ adsorption-desorption isotherms of MCM-41, TiO_2_/MCM-41, and ZTM series samples.

N_2_ adsorption-desorption isotherms were carried out to investigate the textural characteristics of the samples (Figure 10). All the isotherms exhibited the typical type IV corresponding to the mesophases, indicating that incorporation of ZnFe_2_O_4_-TiO_2_ did not destroy the integrity of mesoporous MCM-41 host. The specific surface area (*S*_BET_) and pore wall thickness of ZTM series became smaller than that of MCM-41 (Table 1), confirming the integrated incorporation of ZT nanoparticles inside the MCM-41 pore channels. The reason that the N2 gas absorption at *P*/*P*_0_ = 0 is not zero is attributed to likely existing plentiful micropores, which lead to degassing incompletely.

**Figure 11:**
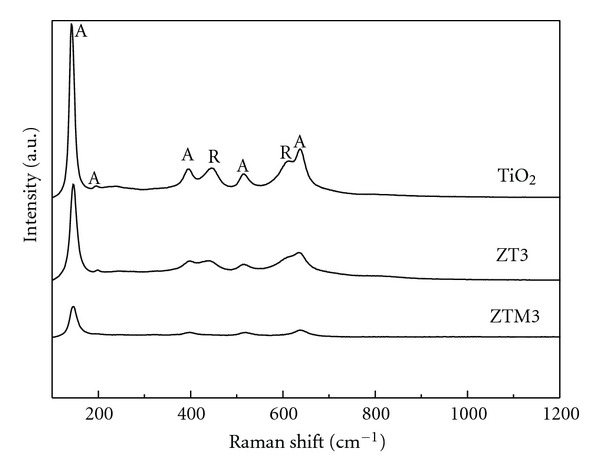
Raman spectra of different samples. (A: anatase, R: rutile.)

Raman spectroscopy is a powerful technique for the investigation of various phases of titanium oxides. Raman spectrum of pure TiO_2_ exhibited the vibration modes of anatase phase at 145, 196, 397, 514, 637 cm^−1^, and rutile phase at 445, 613 cm^−1^ (Figure 11) [41, 42], but no peaks corresponding to Fe_2_O_3_, ZnO, and spinel ZnFe_2_O_4_ was observed in the ZT3 sample, the peaks became broadened asymmetrically with the decrease in intensity. One was attributed to the decrease in particle size [41]; another was the breaking of the symmetry of TiO_2_ molecular structure resulted from the doping of Zn^2+^ and Fe^3+^ ions in the lattice of TiO_2_ [43]. ZTM3 composites only showed the characteristic vibration of anatase, which further demonstrated that MCM-41 can inhibit the phase transformation of TiO_2_, and the broadening of the peaks obviously indicated the smaller particle size of TiO_2_.

**Figure 12:**
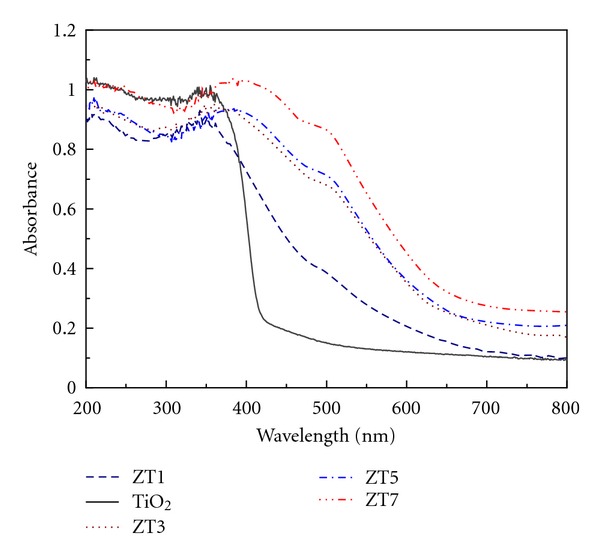
UV-vis spectra of pure TiO_2_ and ZT series samples.

**Figure 13:**
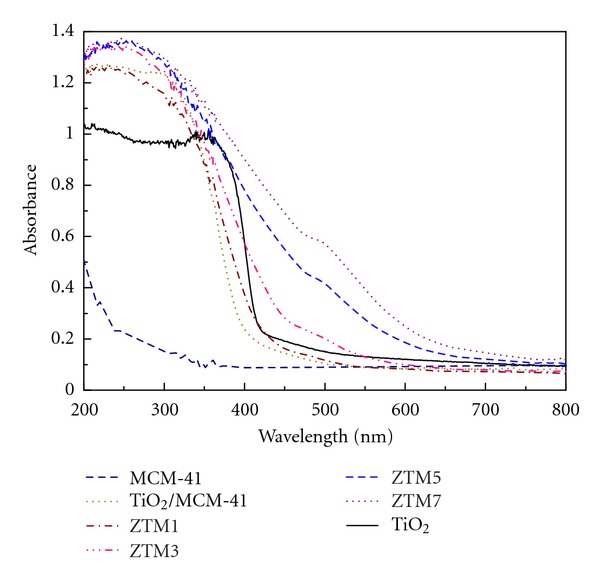
UV-vis spectra of TiO_2_, MCM-41, TiO_2_/MCM-41, and ZTM series samples.

An obvious redshift in ZT samples was observed compared with pure TiO_2_ (Figure 12), the redshift increased regularly with increasing the Zn/Ti molar ratio. The energy gaps were 2.86, 2.19, 1.83, 1.79, and 1.71 eV corresponding to pure TiO_2_ and ZT1, ZT3, ZT5, and ZT7, respectively, the adsorption edges of ZT samples were all in the visible light region, so the addition of ZnFe_2_O_4_ exactly reduced the energy gaps and made the ZT active in visible light [44]. Though an obvious blue shift in MCM-41/TiO_2_ sample was observed compared with pure TiO_2_ (Figure 13), which illustrated that the effect of MCM-41 is that it can be able to control the particle size of TiO_2_ and efficiently prevent particles from agglomeration; Figure 13 further showed an obvious redshift in adsorption edge of ZTM compared with pure TiO_2_, indicating the interesting application of the as-synthesized nanocomposites in the fields of photodegradation and photoelectric devices. The photochemical reactions such as a waste treatment should be developed in the future research.

## 4. Conclusions

ZnFe_2_O_4_-TiO_2_/MCM-41 (ZTM) nanocomposites with different amount of ZnFe_2_O_4_ have been successfully synthesized via a sol-gel method. The addition of ZnFe_2_O_4_ inhibited the growth of TiO_2_ particles and promoted the anatase-rutile phase transformation of TiO_2_. ZnFe_2_O_4_-TiO_2_ nanoparticles would not destroy the pore structure of MCM-41. The ordered pore structure of MCM-41 can effectively control the growth of TiO_2_ nanoparticles. UV-vis absorption edge of ZTM shifted to red regularly with the increase of ZnFe_2_O_4_ and indicated likely excellent visible-light activity. Our present results showed that the as-synthesized ZTM nanocomposite could be a promising multifunctional material in the fields of photodegradation and photoelectric devices.

## Figures and Tables

**Table 1:** Textural characteristics of MCM-41 and ZTM series samples.

Sample	*S*_BET_ (m^2^·g^−1^)	*V* (mL·g^−1^)	*D* (nm)	*d*_100_ (nm)	*a* (nm)	*t* (nm)
MCM-41	830.0	0.64	3.08	3.86	4.46	1.38
ZTM1	657.2	0.49	2.98	3.80	4.39	1.41
ZTM3	838.6	0.64	3.07	3.80	4.39	1.32
ZTM5	655.5	0.46	2.83	3.80	4.39	1.56
ZTM7	724.5	0.52	2.88	3.83	4.42	1.54

*S*_BET_: BET surface area, *V*: pore volume, *D*: average pore diameter, *d*_110_: *d* spacing, *a*_0_: crystal cell parameter, calculated from $a_{0}=2d_{100}/\sqrt{3}$, and *t*: wall thickness (*t* = *a*_0_ − *D*).
